# Consequences of multilingualism for neural architecture

**DOI:** 10.1186/s12993-019-0157-z

**Published:** 2019-03-25

**Authors:** Sayuri Hayakawa, Viorica Marian

**Affiliations:** Department of Communication Sciences and Disorders, Northwestern University, 2240 Campus Drive, Evanston, IL 60208 USA

**Keywords:** Bilingualism, Multilingualism, Neuroplasticity, Experience-dependent plasticity, Language experience, Cognitive function, Executive control, Language learning, Speech processing

## Abstract

Language has the power to shape cognition, behavior, and even the form and function of the brain. Technological and scientific developments have recently yielded an increasingly diverse set of tools with which to study the way language changes neural structures and processes. Here, we review research investigating the consequences of multilingualism as revealed by brain imaging. A key feature of multilingual cognition is that two or more languages can become activated at the same time, requiring mechanisms to control interference. Consequently, extensive experience managing multiple languages can influence cognitive processes as well as their neural correlates. We begin with a brief discussion of how bilinguals activate language, and of the brain regions implicated in resolving language conflict. We then review evidence for the pervasive impact of bilingual experience on the function and structure of neural networks that support linguistic and non-linguistic cognitive control, speech processing and production, and language learning. We conclude that even seemingly distinct effects of language on cognitive operations likely arise from interdependent functions, and that future work directly exploring the interactions between multiple levels of processing could offer a more comprehensive view of how language molds the mind.

## Background

There are nomadic children off the coast of Thailand who can “see like dolphins” [1]. These sea nomads of the Moken tribe spend considerable time diving for food, and have consequently learned to adjust their pupils to improve their vision underwater [2]. Such differences among people of different backgrounds and expertise illustrate the powerful influence that experience can have on the function and physiology of our bodies. What may be more surprising is that experience can change the brain. There is now substantial evidence of neuroplastic changes associated with expertise, ranging from enlarged hippocampi among London taxi drivers [3] to greater volume in insular subregions of expert action video game players [4]. Even brief periods of training have been shown to elicit structural changes, such as in the case of increased gray matter density in the occipito-temporal cortex after just 7 days of learning to juggle [5]. Here, we discuss the neurofunctional and neurostructural consequences of a different type of juggling—namely, the experience of juggling multiple languages within a single cognitive system.

Language processing ranks among the most ubiquitous, yet cognitively complex tasks that we engage in on a daily basis. But unlike the effort put into activities such as practicing the piano or training for a marathon, the pervasiveness of language in almost every facet of our lives makes it easy to overlook as a form of intense exercise. This is especially the case for bilinguals, who may appear to function effortlessly in a single language, while covertly managing multiple linguistic systems that may be competing with each other for activation. Early models of bilingual cognition posited that one language could be independently activated without the other, either through a single “language switch” mechanism (i.e., Penfield and Roberts’ “one switch” model [6]), or through independent switches for output (controlled by the speaker) and input (controlled by the environment) (i.e., Macnamara’s “two switch” model [7]). Since then, research has led to a more integrated view of bilingual cognition, although the strength of activation for each language can indeed be selectively influenced by both top-down (e.g., expectations [8, 9]) and bottom-up inputs (e.g., language-specific acoustic cues [10, 11]). In fact, research utilizing numerous techniques ranging from eye-tracking [12–20] to electroencephalography (EEG) [21–26] has provided ample evidence that multiple languages can be, and often are, activated in parallel. Using eye-tracking and the visual world paradigm [27, 28], Spivey and Marian [12] observed that when Russian-English bilinguals were asked to pick up a particular object from an array, they made eye movements towards other objects with phonologically similar labels. Critically, bilinguals fixated on both within- and between-language competitors, such that an instruction to pick up the “marker” in English would elicit eye movements towards a stamp (“marka” in Russian). This demonstrates that bilinguals may consider lexical candidates from both languages during speech comprehension. Utilizing EEG, Thierry and Wu [26] observed that when Chinese-English bilinguals were asked to judge the semantic relatedness of two words in English, their brain potentials indicated activation of their Chinese translations. Specifically, there was a reduction in the N400 component (an index of semantic integration) both when participants judged words that were related in the target language (English), as well as those that shared a character in the non-target language (Chinese). Evidence of co-activation has been observed across phonological [12], orthographic [29], lexical [21], and morphosyntactic [30] levels of representation, which raises the question of how bilinguals are able to operate in a single-language mode without intrusions from the unintended language.

**Fig. 1:**
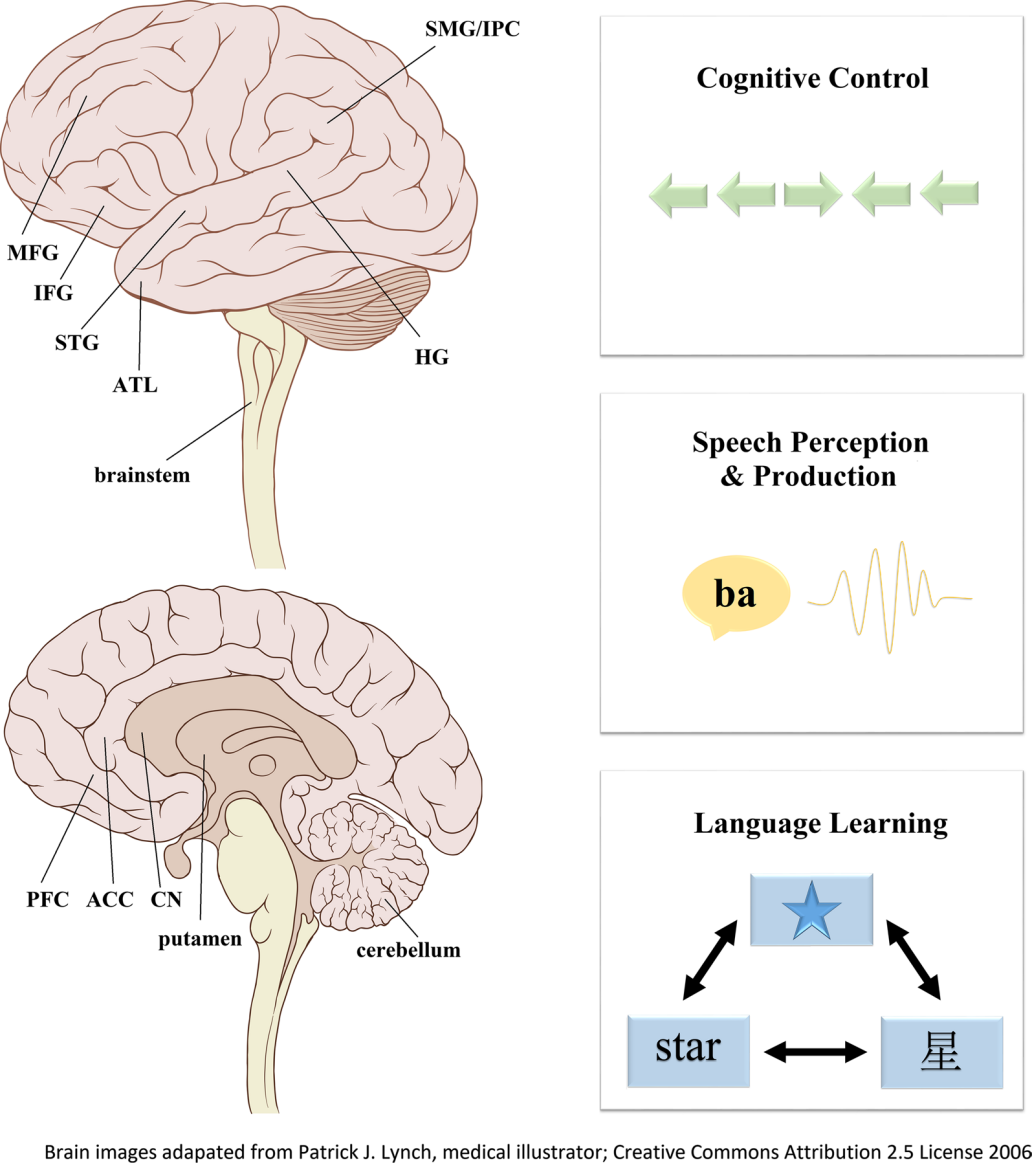
Multilingual experience has widespread consequences for functions ranging from cognitive control to speech processing to language learning. Practice juggling multiple languages leads to functional and structural changes to the brain, such as to the prefrontal cortex (PFC), anterior cingulate cortex (ACC), caudate nucleus (CN), cerebellum, brainstem, Heschel’s gyrus (HG), putamen, superior temporal gyrus (STG), inferior frontal gyrus (IFG), anterior temporal lobe (ATL), and supramarginal gyrus (SMG) in the inferior parietal cortex (IPC)

The precise mechanisms that allow for the successful control of multiple languages have yet to be definitely established. Some have posited that the non-target language is inhibited, others that the target language is facilitated, yet others that the target language is selected (see [31] for a review). Models such as Green and Abutalebi’s Adaptive Control Hypothesis [32] posit a more complex system that includes various functions such as monitoring, inhibition, task engagement and disengagement, which are employed to varying degrees depending on the context. It has also been suggested that bilingual language control may recruit many of the same neural regions utilized for domain-general cognitive control [33, 34]. These include the prefrontal cortex, which is associated with goal maintenance and conflict resolution [34–36], the anterior cingulate cortex and neighboring pre-supplementary motor area, associated with conflict-monitoring and attention regulation [37–39], and the basal ganglia and their constituent regions including the putamen and caudate nucleus, which are associated with functions involved in procedural memory, skill learning, planning, and coordination [38, 40–42]. The repeated engagement of these neural networks to manage language conflict has both functional and structural consequences. In some cases, bilingual experience affects neural activity in the absence of behavioral changes, while in others, it has been associated with a number of language-specific and domain-general advantages relative to monolinguals. In the following sections, we review some examples of how bilingual experience can affect both the function and structure of neural regions underlying different components of language processing. Given that managing language conflict is among the most essential functions for bilingual language processing, we begin with neuroplastic changes to networks associated with linguistic and non-linguistic cognitive control. We then provide evidence that bilingual experience can influence some of the earliest stages of language processing by altering how people encode and attend to sounds, resulting in behavioral consequences for speech perception and production. Lastly, we consider how changes to both high-level executive functions and low-level perception can impact the ability to learn additional languages (e.g., L3, L4, …) (see Fig. 1 for a visual schematic of the processes and neural regions affected by bilingual experience). We broadly organize our discussions around these three topics, not to describe distinct phenomena, but rather to illustrate the ways in which seemingly disparate consequences of bilingual experience may be intertwined through overlapping networks and functions. We therefore conclude by stressing the importance of examining the *relationships* among the various effects of bilingual experience on the brain in order to fully appreciate the widespread and interconnected consequences of living as a multilingual.

## Linguistic and non-linguistic cognitive control

### Functional brain activity

A key feature of bilingual cognition is the parallel activation of multiple languages, and the subsequent need to prevent interference from the non-target language. Because language interference appears to be managed using similar neural networks recruited for general cognitive control, there may be a bilingual advantage for tasks that require ignoring irrelevant information (see [43] for review). Such behavioral differences are most readily observed in children and older adults, while the bilingual advantage appears to be less robust for young adults who generally have a higher capacity for cognitive control [44]. Yet, even when no behavioral differences are observed, there is evidence that bilinguals may be utilizing more efficient control processes. Marian et al. [45] investigated the neural correlates of linguistic control during lexical competition using fMRI and the visual world paradigm described earlier. When monolinguals were asked to select a target among a display that included a phonologically similar competitor, there was significant activation of executive control regions such as the anterior cingulate cortex (ACC) and the superior temporal gyrus (STG) relative to trials without a competitor. Critically, bilinguals did not have significantly greater activation in any regions when resolving within-language competition relative to the control condition. The frequent practice managing competition not only within, but also between languages may make bilinguals more efficient at resolving linguistic conflicts, leading to less reliance on networks associated with cognitive control.

Evidence of more efficient processing has been found during non-linguistic tasks as well. Both Abutalebi et al. [46] and Garbin et al. [47] observed that bilinguals not only outperformed monolinguals during a non-linguistic executive control task, but also had less activity in the ACC, consistent with Marian et al.’s [45] findings. Bilingual experience can additionally influence the functional connectivity between different brain areas. Becker et al. [48] collected fMRI data from bilinguals and monolinguals as they completed a task requiring the application of continuously changing rules. Using Dynamic Causal Modeling, the authors constructed three models depicting the connectivity of three areas known to be associated with cognitive flexibility (ACC, striatum, and dorsolateral prefrontal cortex, or DLPFC) and compared them to the obtained neural data. They observed that for both bilinguals and monolinguals, the ACC was the driving force, influencing activity in the striatum and DLPFC to accomplish tasks involving cognitive flexibility. However, while increased ACC activity resulted in a modest increase in DLPFC and striatum activity for bilinguals, greater ACC activity prompted significant decreases in activity in both regions for monolinguals. The relatively mild influence of the ACC on other regions for bilinguals may be interpreted as a reduced response to conflict, potentially consistent with Abutalebi et al.’s [46] finding. In one case, ACC activity is directly modulated, while in the other, the influence of ACC on other neural structures is reduced.

Studies utilizing EEG have yielded additional evidence that may be indicative of greater neural efficiency among bilinguals [49–52], though with somewhat variable findings depending on the population and task. One commonly examined ERP measure is the N2 component, which is thought to index conflict monitoring [53] or inhibition [54]. The N2 is typically larger when there is a conflict (e.g., incongruent trials of a Simon task) [55], and is correlated with ACC activity [56]. A number of studies have revealed larger N2 amplitudes for bilinguals on conflict trials during Go/No-Go [57, 58] and AX-CPT tasks [52], leading some researchers to conjecture that bilinguals may be engaging in greater conflict monitoring or inhibition. On the other hand, Kousaie and Phillips [51, 59] observed that bilinguals elicited smaller [51] and earlier [59] N2s during a Stroop task compared to monolinguals. While the smaller N2 amplitude among bilinguals differs from the aforementioned findings, it is consistent with the results from fMRI studies observing less bilingual activation of the ACC, which may reflect a reduced need for active conflict monitoring (despite equivalent [51] or even superior [59] performance). It may therefore be the case that depending on the task and population, bilinguals either engage in greater inhibition/monitoring (resulting in larger N2s), or else more efficient general processing, thereby reducing the need for active monitoring (resulting in smaller N2s).

Potentially in line with the latter hypothesis, Kousaie and Phillips observed group differences even for trials without conflicting stimuli (i.e., congruent trials), indicating that bilingualism may confer a global processing advantage (often referred to as the bilingual executive processing advantage, or BEPA [60]). Coderre and van Heuven [61] similarly observed that bilinguals had both faster reaction times and reduced conflict-related ERP amplitudes compared to monolinguals during non-linguistic, non-conflict trials of a modified Stroop task. Group differences in ERP amplitude were even observed before the potentially conflicting target stimulus was presented, suggesting that bilinguals may be engaging in more proactive management of incoming information in the absence of a conflict. However, there is also evidence indicative of greater neural efficiency more specific to active inhibitory control (often described as the bilingual inhibitory control advantage, or BICA [60]). Heidlmayr et al. [62] found that bilinguals using their L2 showed a smaller N400 conflict effect during a Stroop task (i.e., the difference between incongruent and congruent trials) compared to monolinguals. Using a flanker task, Dong and Zhong [49] observed ERP activity consistent with both BEPA and BICA. Relative to bilingual interpreters with less interpreting experience, those with greater experience showed a global processing advantage for conflict monitoring, as indexed by the earlier N2 component (i.e., both congruent and incongruent trials), and more efficient inhibitory control for the later P3 component (i.e., a smaller conflict effect).

Differences in neural efficiency are primarily attributed to experience managing linguistic interference, as mentioned earlier. However, the need to resolve lexical competition is not exclusive to bilinguals, as selecting words within a language also requires the inhibition of semantically and phonologically similar competitors. So why is it that practice resolving lexical conflicts appears to have a more significant impact on domain-general processes for bilinguals than monolinguals? Part of the reason is likely due to the fact that bilinguals experience competition both within and across languages. However, another reason may be because bilinguals utilize more overlapping networks for language processing and domain-general cognitive control relative to monolinguals [47, 63–65]. In one study by Coderre et al. [64], neural activity was measured while participants completed semantic tasks involving non-linguistic competition, linguistic competition, or language processing without competition. The authors observed that while bilinguals recruited similar neural regions for all three tasks (e.g., the left inferior frontal gyrus; L IFG), monolinguals utilized different regions depending on the task. As such, not only do bilinguals have more practice managing linguistic conflict relative to monolinguals, but the impact of such practice on general cognitive control is likely greater as well.

While the exact nature of the mechanisms underlying greater efficiency are still under investigation, some models such as the bilingual anterior to posterior and subcortical shift (BAPSS) model [66] posit that, over time, bilinguals may begin to recruit different regions to manage competition. Specifically, while bilinguals may rely on the typical frontotemporal executive control regions during earlier stages, they may begin to recruit more automatic posterior perceptual/motor areas as they gain greater expertise. Data consistent with this hypothesis include the aforementioned findings that bilinguals rely less on the ACC compared to monolinguals, as well as studies observing greater recruitment of perceptual and motor regions such as the basal ganglia with bilingual experience [67–69]. Luk et al. [70] provide converging evidence by looking at resting-state functional connectivity (assessed by examining the correlations in brain activity between a chosen brain area, the IFG in this case, and all other regions). The bilateral IFG were chosen as the “seeds,” or sources of comparison, because bilinguals in their study had greater white matter integrity in these regions and because the IFG are known to be associated with both language and cognitive control [64]. While monolinguals had stronger associations between the seeds and other frontal regions, bilinguals had stronger associations between the seeds and occipitoparietal regions, supporting the idea that bilingualism may promote the use of more distributed networks involving both frontal and perceptual/motor regions.

In addition to recruiting different networks, bilinguals may have generally greater functional connectivity within and across networks relevant to executive control. Grady et al. [71] found that resting-state functional connectivity was enhanced for bilinguals in the Default Mode Network (DMN; which includes the posterior cingulate, ventromedial prefrontal cortex, angular gyri and parahippocampal gyri), and the frontoparietal control network (FPC). Activity in the DMN is strongest during rest and reduced during externally driven tasks [72]. Greater functional connectivity within the DMN has been shown to promote deactivation during tasks, which in turn facilitates performance [73]. Better executive control is thus predicted by the negative correlation between the DMN and the FPC, the latter of which has highly flexible functional connectivity patterns and facilitates task-specific recruitment of neural regions [74]. In addition to greater functional connectivity within networks, Grady et al. observed that functional connectivity was more correlated across networks for bilinguals relative to monolinguals. In other words, there is evidence that bilingual experience can result in greater and more flexible coordination of different neural regions and networks.

Next, we review evidence that the effects of bilingual experience extend beyond functional changes in neurological activity to the actual structures that support them.

### Structural brain matter

Bilingual experience has been found to increase gray matter density in regions implicated in executive control, including the DLPFC [75], left caudate nucleus (LCN; [40, 76, 77]) and the ACC [78]. As noted previously, the prefrontal cortex, and the DLPFC in particular, is believed to play an important role for domain-general cognitive control [79], as well as language control [35, 80]. Increased gray matter density in regions associated with cognitive control may partly account for the finding that bilingualism can delay the onset of dementia [81, 82]. Consistent with this notion, Abutalebi et al. [78] observed that while both monolinguals and bilinguals experienced age-related gray matter reductions in the DLPFC, reduced gray matter was only correlated with executive control for monolinguals. In other words, while the groups had similar age-related effects at the anatomical level, there were greater negative consequences for monolinguals’ behavioral performance as a result of reduced gray matter. Though no structural differences of the DLPFC were found between the older bilinguals and monolinguals in Abutalebi et al.’s study, Olulade et al. [75] did observe greater gray matter volume among younger, Spanish–English bilinguals compared to monolinguals. However, no such increase was observed for English-ASL bimodal bilinguals. The authors propose that because bimodal bilinguals are able to utilize their two languages simultaneously, language conflict, and subsequent recruitment of the DLPFC, is reduced. Interestingly, bimodal bilingualism has been associated with increased gray matter in the LCN, another region associated with language control [40]. The authors observed that, among bilinguals, there was a positive correlation between gray matter density and LCN activation associated with language switching, providing further support for the involvement of the LCN in bilingual language control. Greater gray matter density for bilinguals compared to monolinguals has additionally been found in the ACC [78], which is associated with conflict monitoring [83, 84]. Abutalebi et al. [46] observed a positive correlation between gray matter in the ACC and both behavioral and functional indices of general cognitive control for bilinguals. Interestingly, no such relationship between gray matter density and functional activation/behavior was observed for monolinguals. This latter result once again suggests that bilingualism can influence both the physical characteristics of neuroanatomical structures, as well as the ways they are utilized.

Potentially related to the issue of processing efficiency, a number of experiments have found a negative relationship between gray matter in the LCN and language exposure/expertise. DeLuca et al. [85] observed that LCN gray matter density of sequential bilinguals was reduced after 3 years of immersion in an L2 context, and Pliatsikas et al. [86] observed differences in the LCN of monolinguals and bilinguals with less, but not more immersive experience. Similarly, Elmer et al. [87] found that highly trained simultaneous interpreters had less gray matter volume in several language control regions compared to multilingual non-interpreters, and that gray matter in the bilateral caudate nucleus was negatively correlated with the number of interpreting hours. At first glance, these results seem at odds with the general observation that gray matter increases with greater language competence (e.g., Hervais-Adelman et al. [76] who observed a positive relationship between gray matter in the caudate nucleus and a composite index of multilingual experience). However, as speculated by Elmer et al. [87], reductions in gray matter may reflect cortical pruning associated with greater specialization and efficiency. In other words, gray matter density in particular regions (such as the LCN) may initially increase as bilinguals gain greater mastery over their languages, but then decrease as they become more efficient at carrying out necessary functions (such as reducing interference from unwanted languages). This greater efficiency could result from a number of different mechanisms, including increased specialization of a particular region (such as the ACC as suggested by Abutalebi et al. [46]) or else reliance on regions associated with different, potentially more procedural, functions (consistent with the previously discussed BAPSS model [66]). For instance, DeLuca et al. [85] observed that the same population of bilinguals who experienced a reduction in the LCN had significantly increased gray matter volume in the cerebellum. Increased gray matter in the cerebellum has been associated with the ability to control interference from a non-target language [88], as well as grammatical processing in bilinguals [89]. DeLuca et al. propose that their pattern of results may reflect a shift in neural networks as a result of more automated L2 processing.

As noted previously, neuroimaging and electrophysiological evidence suggest that bilinguals may rely on more distributed networks compared to monolinguals [47, 65]—a conclusion further supported by studies examining the integrity of white matter tracts connecting different areas of the brain. When comparing older bilingual and monolingual adults, Luk et al. [70] found that bilinguals had higher fractional anisotropy (FA) values, an indirect measure of white matter integrity, in the corpus callosum (CC; see also [90, 91]), extending to bilateral superior longitudinal fasciculi (SLF; see also [91]), and the right inferior fronto-occipital fasciculus (IFOF; see also [91–93]). The CC is a thick tract connecting the left and right hemispheres, and is associated with high-level cognitive processes such as executive function [94, 95]). The SLF is a long-range tract connecting the frontal lobe to posterior parietal and temporal cortices, which along with the arcuate fasciculus (AF) is often classified as the dorsal stream of the language network (especially implicated in speech perception and production [96]). The IFOF connects frontal, occipital, and parietal cortices, and has been proposed as the ventral stream of language processing (associated with semantic processing [97]). Bilinguals with greater white matter integrity have also demonstrated greater functional connectivity between frontal and posterior cortical regions [70]. In other words, bilingual experience can facilitate more distributed functional connectivity, likely supported by the integrity of white matter structures connecting the frontal lobe with more distant brain areas.

While a number of studies have reported greater white matter integrity for bilinguals compared to monolinguals, particularly in the IFOF [91–93], there is also evidence of the opposite pattern [98–100]. For instance, Gold et al. [99] observed that compared to age-matched monolinguals, older bilinguals had less white matter integrity in a number of tracts, including the IFOF, CC, and fornix (which originates in the hippocampus and is associated with memory function [101]). Despite the apparent inconsistency with Luk et al’s findings [70], the authors point out that the bilinguals’ cognitive functioning did not differ from monolinguals despite lower white matter integrity. In fact, behavioral and fMRI data from the same subjects showed that the bilinguals were faster at task-switching despite less activation in frontal executive control regions [99]. The authors thus propose that bilinguals may be efficiently compensating for reduced integrity in some tracts through the use of different pathways and neural regions (such as the relatively intact SLF connecting frontal and subcortical areas in the executive network).

Practice learning and managing multiple linguistic systems thus influences how individuals resolve conflict, in some cases, leading to what appears to be more efficient cognitive control. Table 1 provides a summary of studies of language effects for tasks and regions relevant to linguistic and non-linguistic cognitive control. As can be seen, bilinguals often have less activation of cortical regions traditionally associated with cognitive control (such as the ACC and the PFC) when managing conflict. On the other hand, in addition to greater gray matter volume in these same frontal regions, bilinguals often have stronger activation of control-relevant subcortical areas (e.g., LCN), which is likely facilitated by more widespread and flexible functional and structural connectivity. These patterns reflect two possible ways in which bilingual experience may support executive function—by enhancing the robustness of the underlying neural structures, as well as by potentially recruiting more efficient networks to accomplish the same cognitive control task.

**Table 1:** Consequences of bilingualism for linguistic and non-linguistic cognitive control

	Type	Region	Effect	Task	Study
ACC	Functional	ACC	Mono ≠ bi (greater activation for switch than non-switch for mono only)	Non-verbal task switching	Garbin et al. [47]
		ACC	Mono ≠ bi (greater activation for competitor than control, mono only)	Visual world (phonological competition)	Marian et al. [45]
		ACC	Mono > bi (activation associated with conflict effect)	Flanker	Abutalebi et al. [46]
		ACC	Mono > bi (activation associated with conflict effect)	Stroop	Waldie et al. [69]
	Structural	ACC	Mono ≠ bi (− correlation: gray matter/conflict effect, bi only)	Flanker	Abutalebi et al. [46]
		ACC	Bi > mono (gray matter)	Flanker	Abutalebi et al. [78, 179]
		ACC	Multilingual controls > interpreters (gray matter); controls ≠ interpreters (− correlation: gray matter/interpreting hours; interpreters only)		Elmer et al. [87]
Frontal cortex/gyrus	Functional	L IFG	Mono ≠ bi (overlapping activation across tasks, bi only)	Linguistic/non-linguistic flanker; semantic categorization	Coderre et al. [64]
		L IFG	Mono ≠ bi (greater activation for switch than non-switch, bi only)	Non-verbal task switching	Garbin et al. [47]
		R IFG	Mono ≠ bi (greater activation for switch than non-switch, mono only)	Non-verbal task switching	Garbin et al. [47]
		SFG	Mono ≠ bi (greater activation for competitor than control, mono only)	Visual world (phonological competition)	Marian et al. [45]
		SFG, MFG, IFG	Mono > bi (activation associated with conflict effect)	Stroop	Waldie et al. [69]
		R SFG/R MFG	Between > within-language (activation associated with conflict effect)	Visual world (phonological competition)	Marian et al. [67]
		R SFG/R MFG/R IFG	Dominant > non-dominant language competition (activation associated with conflict effect)	Visual world (phonological competition)	Marian et al. [67]
	Structural	DLPFC	Mono ≠ bi (− correlation: gray matter/conflict effect, mono only)	Flanker	Abutalebi et al. [78, 179]
		SFG	Bi > mono (gray matter)	Language switching	Zou et al. [40]
		MFG, IFG, R SFG	Bi > mono (gray matter)		Olulade et al. [75] (experiment 1)
		IFG	Multilingual controls > interpreters (gray matter); controls ≠ interpreters (− correlation: gray matter/interpreting hours; interpreters only)		Elmer et al. [87]
Temporal cortex/gyrus	Functional	MTG, STS	Mono ≠ bi (greater activation for competitor than control, mono only)	Visual world (phonological competition)	Marian et al. [45]
	Structural	R MTG, R ITG,	Mono > bi (gray matter)		Olulade et al. [75] (experiment 1)
		R STG, L MTG	Bi > mono (gray matter)		Olulade et al. [75] (experiment 1)
		L ITG	Bi > mono (gray matter)	Language switching	Zou et al. [40]
Parietal cortex/gyrus	Functional	L IPL	Mono ≠ bi (greater activation for switch than non-switch for mono only)	Non-verbal task switching	Garbin et al. [47]
	Structural	R IPL	Bi > mono (gray matter)		Olulade et al. [75] (experiment 1)
		L SMG	Multilingual controls > interpreters (gray matter)		Elmer et al. [87]
Occipital cortex/gyrus	Functional		Bi > mono (EEG complexity); mono ≠ bi (− correlation: complexity/conflict effect, bi only)	Non-verbal task switching	Grundy et al. [66, 68]
	Structural	L SOG, L IOG	Bi > mono (gray matter)		Olulade et al. [75] (experiment 1)
Subcortical	Functional	L CN	Bi > mono (activation associated with conflict effect)	Stroop	Waldie et al. [69]
		L CN	Bi only: more LCN activation when language switching than when not	Language switching	Zou et al. [40]
		L CN/L putamen	Bi only: between > within-language (activation associated with conflict effect)	Visual world (phonological competition)	Marian et al. [67]
	Structural	L CN	Bi > mono (gray matter)	Language switching	Zou et al. [40]
		L CN	More > less multilingual experience (gray matter)		Hervais-Adelman et al. [76]
		Striatum	Mono ≠ bi (+ correlation: gray matter/faster switching, bi only)	Non-verbal task switching	Garbin et al. [47]
		CN	Controls ≠ interpreters (− correlation: gray matter/interpreting hours; interpreters only)		Elmer et al. [87]
		L CN/Hip/Amg	Less > more immersion (gray matter contraction)		Deluca et al. [85]
		L Cb	More > less immersion (gray matter)		Deluca et al. [85]
		Cb	Mono > bi (gray matter)		Olulade et al. [75] (experiment 1)
Multiple/other	Functional	ACC, PFC, striatum	Bi ≠ mono (− correlation: ACC/PFC and striatum for monos; + correlation: ACC/PFC and striatum for bis)	Rapid instructed task learning	Becker et al. [48]
		e.g., ACC, PFC, CN, putamen	Bi > mono (overlapping activation across tasks)	Verbal/non-verbal switching	Anderson et al. [63]
			Bi > mono (overlapping activation across tasks)	Verbal/non-verbal task switching	Timmer et al. [65]
			Bi < mono (amplitude of N2 in Stroop), mono > bi (amplitude of P3 in Simon), bi ≠ mono (longer delay in P3 latency in Eriksen in monos)	Stroop, Simon, and Eriksen tasks	Kousaie and Phillips [51]
			Bi < mono (N_Inc_ positivity post-target onset) Bi > mono (N_Inc_ negativity pre-target onset)	Stroop	Coderre and van Heuven [61]
			Bi < mono (N400 conflict effect for Stroop)	Stroop/negative priming	Heidlmayr et al. [62]
			Bi > mono (CRN and ERN negativity)	LANT	Kałamała et al. [50]
			More > less interpreting experience (N1/N2 amplitude); less > more interpreting experience (P3 amplitude for incongruent trials only)	Flanker	Dong and Zhong [49]
			Bi > mono (N2 on NoGo)	Go/NoGo	Fernandez et al. [57]
			Bi > mono (N2 and late positivity wave on NoGo)	Go/NoGo	Moreno et al. [58]
			Bi > mono (N2 and P3 to AY)	AX-CPT	Morales et al. [52]
		DMN, FPC	Bi > mono (resting-state connectivity within and between networks)		Grady et al. [71]
		Frontal, occipital, parietal regions	Bi > mono (frontal-occipitopartietal resting-state connectivity) Bi < mono (frontal resting-state connectivity)		Luk et al. [70]
	Structural	SLF, IFOF	Bi > mono (white matter)		Luk et al. [70]
		ILF/IFOF, fornix, CC	Mono > bi (white matter)		Gold et al. [99]

Summary of functional and structural effects of bilingual experience for tasks and neural regions associated with linguistic and non-linguistic cognitive control.

Given the pervasive involvement of cognitive control in a wide variety of tasks (e.g., [102–104]), an effect of bilingualism on this central function could initiate a chain of consequences across multiple domains and stages of processing. We illustrate the potentially vast impact of bilingual effects on the brain by considering one of the earliest stages of language processing: the perception and production of speech sounds.

## Speech perception and production

### Functional brain activity

Studies utilizing EEG have provided evidence that bilingual experience can enhance attention to speech stimuli [105–107]. For instance, both bilingual toddlers and bilingual adults are quicker than monolinguals at detecting language switches [105, 106]. Further evidence comes from a study in which children were presented with pictures followed by words that were either related or unrelated. While ERPs did not vary between language groups at later stages of semantic processing, only bilinguals had ERPs indicating attention to unexpected phonemes during early stages [107]. In addition to earlier responses to speech stimuli, Chinese–English bilinguals have been found to attend more globally to entire words as compared to English monolinguals who focus the most on word onsets, as indexed by the relative amplitude of the N1 component, which is associated with attention [108]. The authors suggest that because the segments of words that are most critical for word identification may vary across different languages, it may be more efficient to distribute attention across all segments rather than switch strategies depending on which language is being used. In other words, bilingual experience leads to changes in both the time course and distribution of attentional allocation, and has been found to enhance attentional control when processing non-speech stimuli as well (e.g., tones [109]).

Enhanced attentional control among bilinguals has even been associated with changes to how robustly speech sounds are encoded at the level of the brainstem [110–115]. Auditory brainstem responses (ABR) are a measure of encoding strength, and encoding of the fundamental frequency (f0) in particular has been found to be both experience-dependent [116, 117] and predictive of speech perception ability [118, 119]. Krizman et al. [111] observed that encoding of the f0 was more robust for bilinguals than monolinguals when listening to speech stimuli such as/da/, and that this enhancement was correlated with attentional control. This finding highlights the potential interconnectivity of bilingual effects on executive function and speech-sound processing. Furthermore, the relationship between the consistency of ABRs and attentional control has been found to be stronger among bilinguals compared to monolinguals, likely as a result of the greater demands associated with communicating in multiple languages [110]. Recent work has revealed that the effect of bilingualism on neural encoding is remarkably consistent, with similar effects regardless of socioeconomic status [113], and for bilingual speakers of more than a dozen languages [115]. Moreover, despite the fact that monolingual speakers of tone languages such as Cantonese and Mandarin have been shown to have highly robust brainstem responses [117, 120], bilingual speakers of two tone languages exhibit even stronger encoding [114]. This additive effect of language experience aligns with the finding that the consistency and strength of f0 encoding among children is positively associated with the amount of bilingual experience [112].

A recent study by Zhao and Kuhl [121] confirmed the link between brainstem encoding and conscious speech perception. Building on the well-established finding that the perception of speech sounds varies as a function of native language background (e.g., sensitivity to phonemic contrasts of the native language [122]), the authors observed that differences in how sounds were perceived correlated with different patterns of encoding at the brainstem. It is possible that more robust and consistent encoding among bilinguals could additionally facilitate discrimination of non-native contrasts, as has been found for individuals with musical expertise [123]. Early in development, infants are sensitive to phonetic contrasts of all languages, but eventually become tuned to their native language. However, it has been suggested that bilingual experience may prolong the period of universal discriminability (i.e., “the perceptual wedge hypothesis”). Petitto et al. [124] found that while 10 to 12-month-old monolingual infants were no longer sensitive to non-native contrasts, activity in the LIFC indicated that bilingual infants were sensitive to phonetic contrasts of both native and non-native languages. Findings from a recent MEG study additionally suggest that 10 to 12-month-old bilinguals may analyze speech sounds based on acoustic (as opposed to phonetic) properties to a greater extent than monolinguals [125]. This prolonged period of acoustic analysis may be adaptive for dealing with the increased variability associated with multiple phonemic systems and may help bilinguals retain the ability to discriminate non-native contrasts. Indeed, bilingual infants as old as 18–20 months were found to be sensitive to the phonemic contrasts of a novel language (Ndebele clicks), while monolinguals were not [126].

While enhanced bilingual discrimination of non-native contrasts does not appear to persist into adulthood at initial exposure [127, 128], there is evidence suggesting that bilinguals may be better at learning non-native contrasts relative to monolinguals after training [127, 129, 130]. In addition to better discrimination of non-native contrasts during comprehension, bilingualism may confer advantages for the production of novel sounds. For example, Spinu et al. [131] recently found that after training, bilinguals were better able to reproduce a non-native Sussex English accent (as measured by the glottal-stop rate) compared to monolinguals. The authors conjecture that this bilingual advantage for phonological acquisition may be related to their more robust encoding of speech sounds at the level of the brainstem. Specifically, they propose that stronger subcortical encoding of sounds may translate to richer acoustic signals in auditory sensory memory, eventually leading to more efficient processing of speech.

As with executive control, speech perception and production are influenced not only by the efficiency of particular neural regions, but also by the functional connectivity across brain areas. For instance, Ventura-Campos et al. [132] discovered that resting-state functional connectivity between inferior frontal (left frontal operculum/anterior insula) and parietal regions (left superior parietal lobule) predicted how well individuals were able to learn non-native contrasts after training. As noted previously, bilingualism has been shown to enhance resting-state functional connectivity in the frontal–parietal network [71]. Additionally, it has been found that early bilinguals have greater functional connectivity in language networks relevant to phonological processing (as well as semantic processing) relative to late bilinguals with comparable proficiency [133]. Berken et al. [134] similarly observed that early bilinguals had greater connectivity between the IFG and a number of language processing and executive control regions including the cerebellum, which is associated with processes underlying speech perception and production [135], as well as language control [88].

### Structural brain matter

Bilingual experience has been associated with structural changes to brain regions supporting both auditory processing and speech production. Ressel et al. [136] observed a relationship between early bilingual experience and increased gray and white matter volumes in Heschel’s gyrus (HG), a part of the temporal lobe that contains the primary auditory cortex. Faster [137] and more successful [138] identification of foreign speech sounds has been linked to greater white and gray matter volumes, respectively. Enhanced volume in this region among bilinguals thus coincides with the aforementioned advantages for learning non-native phonemic contrasts [127, 129, 130]. Mårtensson et al. [139] observed that compared to controls, bilingual interpreters had increased cortical thickness in the superior temporal gyrus (STG), a region consistently activated during acoustic–phonetic processing [140]. The authors additionally found greater cortical thickness among interpreters in the middle frontal gyrus (MFG), part of the articulatory network that contributes to pronunciation aptitude [141]. A recent study with bilingual children found that stronger foreign accents were associated with a reduction in the surface area of the STG and MFG [142], though balanced bilingual children had relatively less cortical thickness in these regions. Rodriguez et al. [143] similarly found that cortical thickness of the anterior insula was negatively correlated with the ability to learn foreign phonological contrasts among bilinguals. These latter results may originate from similar processes as the previously-discussed reduction in gray matter volume for expert interpreters [87]. Specifically, it may be the case that initial training enhances cortical volume/thickness while greater expertise and efficiency will eventually lead to cortical pruning. Consistent with this notion, Elmer et al. [144] found that interpreters had significantly reduced white matter relative to controls in regions associated with sensory-motor coupling and speech articulation (e.g., L anterior insula, R IPL, and upper cortico-spinal tract), as well as with executive function (e.g., R CN, CC).

One region that has been found to be larger for balanced bilingual children is the putamen [142], consistent with a number of other studies finding greater gray matter density in this region for bilinguals compared to monolinguals [41, 86, 145]. The putamen has been implicated in language production and articulation [146, 147], dramatically evinced by the fact that lesions to the area can disrupt a speaker’s ability to produce phonemes of their native language, resulting in speech that appears to be foreign accented (i.e., Foreign-Accent Syndrome [148]). Greater gray matter density in the putamen of bilinguals compared to monolinguals is likely to result from the more complex articulatory repertoire associated with learning and utilizing multiple languages. Among bilinguals, lower proficiency has been associated with greater putamen activity, possibly reflecting increased articulatory effort [41, 146, 149]. Additionally, Berken et al. [150] observed that among sequential bilinguals, there was a positive correlation between more native-like accents and gray matter density in the left putamen (as well as a number of other regions implicated in speech-motor control). Together, these findings may suggest that gray matter density in the left putamen underlies native-like articulation ability, and that speakers experiencing difficulty may compensate by activating this region to a greater degree (potentially inducing structural changes as they improve).

Lastly, and as noted previously in our discussion of cognitive control, bilingual experience has been shown to enhance white matter integrity in the SLF [70, 77, 91], as well as the IFOF [91–93], representing the dorsal and ventral streams of language processing, respectively. The SLF, and the AF in particular, are associated with phonological processing and articulation [151]. Among bilinguals, a number of variables have been shown to affect white matter integrity in the SLF/AF. Higher proficiency [152] and greater immersive L2 experience [100, 153] are predictive of enhanced structural integrity, while findings for age of L2 acquisition are mixed [152, 154]. While Nichols and Joannis [152] found a positive association between age of acquisition and white matter in the AF, Hämäläinen et al. [154] observed greater white matter for early compared to late bilinguals.

Table 2 summarizes recent research on the functional and structural effects of multilingual experience on operations and brain regions relevant to speech perception and production. There is reliable evidence that bilingual experience can enhance attention to speech stimuli and result in more consistent and robust encoding of sound in the brainstem, suggesting that bilingual experience has (often beneficial) effects on the neural functions underlying both cognitive control and speech processing. Furthermore, the consequences of bilingual experience on speech processing may partly originate from changes to cognitive control. Bilingualism has also been found to affect the structure of regions underlying both functions, though in some cases, it is not yet clear how specific anatomical characteristics align with behavioral expertise and outcomes. In the final section, we extend the potential “chain of bilingual consequences” one step further by exploring how enhanced cognitive control and speech processing may translate to a greater capacity for learning new languages.

**Table 2:** Consequences of bilingualism for speech perception and production

	Type	Region	Effect	Task	Study
ACC	Functional	ACC/SMA	Lower > higher proficiency (activation associated with reading)	Single word reading	Meschyan and Hernandez [149]
Frontal cortex/gyrus	Functional		Mono ≠ bi (N1 amplitude; largest at word onset, mono only)	Speech perception	Astheimer et al. [108]
			Mono ≠ bi (P2 positivity in response to language change, bi only; P2 positivity greater for matching than mismatching stimuli, bi only)	Picture-word relatedness (matching vs. mismatching)	Kuipers and Thierry [105]
		L IFC	Bi > mono (activation)	Speech perception	Petitto et al. [124]
	Structural	R DLPFC	Simultaneous > sequential bilinguals (gray matter)		Berken et al. [134, 150]
		MFG/IFG	Interpreters > control (cortical thickness after training). interpreters ≠ control (+ correlation MFG cortical thickness/effort; interpreters only)	Proficiency test	Mårtensson et al. [139]
		L MFG/L IFG	Unbalanced > balanced (cortical thickness); unbalanced ≠ balanced (− correlation foreign accent/MFG surface area)	Proficiency test	Archila-Suerte et al. [142]
Temporal cortex/gyrus	Structural	L STG	Unbalanced > balanced (cortical thickness); unbalanced ≠ balanced (− correlation foreign accent/STS surface area)	Proficiency test	Archila-Suerte et al. [142]
		STG	Interpreters > control (cortical thickness after training). interpreters ≠ control (+ correlation STG cortical thickness/proficiency; interpreters only)	Proficiency test	Mårtensson et al. [139]
		HG	Bi > mono (HG volume, gray matter)		Ressel et al. [136]
Parietal cortex/gyrus	Structural	R IPL	Interpreters < control (white matter)		Elmer et al. [87]
Occipital cortex/gyrus	Structural	L MOG/R LOC	Simultaneous > sequential bilinguals (gray matter)		Berken et al. [134, 150]
Subcortical	Functional	Brainstem	Bi > mono (ABR consistency); mono ≠ bi (+ correlation: ABR/selective attention, bi only)	Sustained selective attention/speech perception	Krizman et al. [111]
		Brainstem	Bi > mono (ABR consistency); mono ≠ bi (+ correlation: ABR/attentional control and proficiency, bi only)	Attentional control/speech perception	Krizman et al. [110]
		Brainstem	Bi > mono (ABR consistency)	Speech perception	Krizman et al. [113]
		Brainstem	Bi > mono (FFR)	Speech perception	Skoe et al. [115]
		Brainstem	Bi > mono (FFR)	Speech perception	Maggu et al. [114]
		Brainstem	Simultaneous > sequential bilinguals (ABR consistency; + correlation bilingual experience/ABR)	Speech perception	Krizman et al. [112]
		L putamen	L3 > L2 (activation associated with picture naming)	Picture naming	Abutalebi et al. [41]
		Putamen	Greater > lower proficiency (activation)	Single word reading	Meschyan and Hernandez [149]
	Structural	Putamen, thalamus, R CN, L globus pallidus	Bi > mono (gray matter expansion)		Burgaleta et al. [145]
		putamen	Balanced > unbalanced (volume)	Proficiency test	Archila-Suerte et al. [142]
		L putamen	Simultaneous > sequential bilinguals (gray matter); simultaneous ≠ sequential bilinguals (+ correlation: gray matter/native-like accent; sequential only).		Berken et al. [134, 150]
		L putamen	Bi > mono (gray matter); bi ≠ mono (+ correlation: gray matter/L3 proficiency; bi only)	Picture naming	Abutalebi et al. [41]
		R Hip	Interpreters > control (volume after training). interpreters ≠ control (+ correlation Hip volume/proficiency; interpreters only)	Proficiency test	Mårtensson et al. [139]
		Upper corticospinal tract, R CN	Interpreters < control (white matter)		Elmer et al. [144]
Multiple/other	Functional		Bi > mono (ERP amplitude)	Selective attention to tones	Rämä et al. [109]
			Bi > mono (early MEG activity showing more acoustic processing of speech stimuli)	Oddball paradigm (sound perception)	Ferjan Ramírez et al. [125]
			Bi > mono (bilinguals faster at differentiating languages, as indicated by ERP)	Oddball paradigm (picture-word pairs)	Kuipers and Thierry [106]
			Mono ≠ bi (early ERP positivity for semantically matching pictures/words, bi only)	Oddball paradigm (picture-word pairs)	Kuipers and Thierry [107]
		IFG, DLPFC, IPL, cerebellum	Early bi > late bi (resting functional connectivity); + correlation (AoA and connectivity between L/R IFG for late bi)	Speech production task	Berken et al. [134, 150]
		semantic module (seed: L SMG); phonological module (seed: L IFG_pt_)	Early bi > late bi (resting functional connectivity in both modules)		Liu et al. [133]
	Structural	CC, IFOF, uncinate fasciculi (UF), SLF	Bi > mono (white matter)		Pliatsikas et al. [91]
		SLF, L IFOF, L UF	+ Correlation (L2 listening experience and white matter in UF and anterior IFOF); + correlation (L2 speaking experience and white matter in posterior SLF and IFOF); bi only; mono > bi (white matter)		Kuhl et al. [100]
		CC, cingulum, AF, L IFOF	Bi > mono (white matter); + correlation (immersion time and white matter in all but cingulum; bi only)		Rahmani et al. [153]
		ILF, CC, AF	+ Correlation (AoA with L IFL, anterior CC, AF); + correlation (proficiency with L ILF, R AF, forceps minor of CC)	Picture-word matching	Nichols and Joanisse [152]
		AF	Early bi > late bi (white matter)		Hämäläinen et al. [154]
		CC, L anterior insula	Interpreters < control (white matter)		Elmer et al. [144]

Summary of functional and structural effects of bilingual experience for tasks and neural regions associated with speech perception and production.

## Language learning

### Functional brain activity

Evidence suggests that bilingual experience may confer benefits for learning new languages beyond the two that are already known, in part, as a result of changes to executive function [155–160] (see [161] for review). For example, Kaushanskaya and Marian [159] observed that bilinguals were better able to learn novel words that had letter-to-sound mappings that diverged from those of their known languages. Participants were asked to recall words after hearing a novel word from an artificial language either with or without its written form. In cases where participants read a word with orthography that conflicted with letter-to-sound mappings of known languages, bilinguals were significantly better at inhibiting interference from letter-to-sound mappings of their native tongue, thereby outperforming monolinguals. Similarly, Bartolotti and Marian [155] observed that when participants completed a visual world task and were asked to select novel words with phonological overlap with a known language, monolinguals looked more at native language competitors relative to bilinguals. In addition to vocabulary acquisition, there is evidence that bilingualism may facilitate learning of novel syntax [162, 163], though the findings are somewhat mixed. In a recent EEG study, Grey et al. [164] observed no behavioral difference between bilinguals and monolinguals learning an artificial language. However, there were distinct ERP patterns associated with their grammaticality judgments. At high proficiency, both monolinguals and bilinguals showed native-like ERPs (a P600 component associated with syntactic processing), whereas at low proficiency, only bilinguals showed this pattern. In other words, even when no behavioral differences are observed, bilinguals show more native-like processing at early stages of acquiring a novel language. The authors suggest that enhanced cognitive control could once again play a role, as bilinguals may be better able to reduce interference from the syntax of known languages. As noted previously, this greater efficiency may be achieved through the recruitment of different neural networks to control interference from a non-target language. Evidence of such a process during language learning comes from Bradley, King, and Hernandez [165] who found that after just 2 h of exposure to a new language, bilinguals were not only faster at making semantic judgments in response to novel words compared to monolinguals, but also recruited different neural networks. Specifically, monolinguals relied more on regions typically associated with executive control such as the DLPFC, ACC, SMA, and LCN, while bilinguals only showed increased activation in the putamen.

Given that the putamen is associated with phonological processing and articulation [166, 167], the bilingual advantages for language learning may be connected to the effects of language experience on speech processing, in addition to cognitive control. Consistent with this notion, Kaushanskaya and Marian [157] found that bilinguals outperformed monolinguals when learning novel words, and that better performance was correlated with phonological working memory among early bilinguals, but not late bilinguals or monolinguals. This was despite the fact that phonological working memory has been previously linked to word learning for monolinguals [168, 169]. The authors propose that because the novel words were phonologically dissimilar to the native language (English), monolinguals and late bilinguals may not have been able to efficiently utilize their phonological working memory to learn them. Early bilinguals, on the other hand, may be able to utilize phonological working memory resources more efficiently to learn even non-native-like words. Recall that Spinu and colleagues [131] proposed a similar hypothesis, suggesting that more robust subcortical sound encoding could increase the availability of acoustic signals in auditory sensory memory. This could in turn allow for more effective recruitment of phonological working memory, facilitating discrimination of unfamiliar phonetic contrasts, and potentially, the acquisition of novel vocabulary.

### Structural brain matter

Bilingual experience has been shown to elicit structural changes in regions that support language processing and acquisition. The first discovery of neurostructural changes as a result of bilingual experience was reported by Mechelli et al. [170], who observed increased gray matter density in the posterior supramarginal gyrus (pSMG; located in the LIPC) for bilinguals compared to monolinguals. The LIPC has been associated with a number of functions relevant to language learning, including the maintenance of mental representations, verbal and phonological working memory, the integration of semantic and phonological information, and cognitive control [171–176]. Extending Mechelli et al.’s [170] finding, Grogan et al. [177] observed increased gray matter in the pSMG for multilinguals of three or more languages relative to bilinguals, indicating that experience-dependent changes to this region can vary by the degree of multilingualism in addition to categorical differences between monolinguals and bilinguals. Indeed, gray matter density in the LIPC has been shown to be negatively correlated with age of acquisition and positively correlated with language competence among bilinguals [170, 178]. Furthermore, Della Rosa et al. [178] observed that the amount of gray matter was positively associated with cognitive control, as assessed by an Attentional Network Task. Consistent with the previously discussed neuroprotective function of bilingualism, Abutalebi et al. [179] found that while monolinguals displayed age-related gray matter reductions in the right inferior parietal lobule, no age-related decline was observed for bilinguals.

There is also evidence of greater gray matter volume for older bilinguals compared to monolinguals in the temporal pole [180], which for bilinguals, is positively correlated with the ability to name pictures in L2 [181]. Grogan et al. [177] similarly observed a positive correlation between gray matter density in the LIFG and lexical efficiency (as assessed by a lexical decision task), as well as a negative correlation between gray matter and age of acquisition. While language is generally associated with left-lateralized regions, Hosoda et al. [77] found that L2 vocabulary size was positively correlated with gray matter density in the right IFG, as well as greater white matter integrity in a number of language-related networks. García-Pentón et al. [182] similarly observed that bilinguals have greater structural connectivity in a number of networks that support language processing, including the IFG, SFG, and STG, which have been associated with managing phonological, syntactic, and semantic interference between languages. Greater functional connectivity among similar regions (as well as the DMN) has been shown to predict how well individuals are able to learn novel words [183]. Given that bilinguals were found to have greater connectivity within the DMN as well [71], structural and functional changes associated with knowing multiple languages could potentially facilitate the acquisition of additional languages.

As previously discussed, one of the most consistently observed effects of bilingualism on white matter integrity is in the IFOF [70, 92, 93, 152, 154], the ventral stream of the language processing network implicated in the semantic processing of language [184]. Nichols and Joanisse [152] found that, among bilinguals, age of acquisition and proficiency were independently correlated with FA values in different tracts. While age of acquisition was uniquely and positively associated with bilateral inferior longitudinal fasciculi (ILF) and otherwise primarily left-lateralized regions, such as of the CC and the AF, proficiency was uniquely and positively associated with corresponding right-lateralized regions. The authors conjecture that the former may reflect the increased effort of utilizing an L2 learned later in life, while the latter may indicate greater efficiency resulting from mastery over the language. They additionally point out that the relationship between white matter integrity and proficiency may either be causally related (such that greater proficiency leads to the development of more robust white matter tracts), or else that certain individuals may be predisposed to both greater proficiency and the development of higher white matter integrity. Similarly ambiguous is whether there is indeed a causal relationship between the effects of bilingual experience on structural changes and the ability to acquire new languages (e.g., L3, L4). Determining the nature of this relationship is particularly difficult when comparing life-long bilinguals and monolinguals, as the two groups naturally vary in a number of ways other than language experience and neural structures. Establishing the causal links between (1) language experience and changes to neural structures, and (2) neural structures and language learning will likely require more longitudinal research, as well as controlled experiments that explicitly manipulate language experience and track outcomes for later learning.

While few studies have employed true random assignment to manipulate language experience coupled with assessments of later language learning, there are several longitudinal studies examining pre- and post-training correlates of language ability [77, 139, 185]. Stein et al. [185] tested English-speaking exchange students learning German on day 1 of their stay in Switzerland as well as 5 months later when proficiency was significantly increased. They observed a significant positive correlation between proficiency and gray matter density in the IFG, but no relationship between the absolute values of density and proficiency at either time point. Hosoda et al. [77] similarly observed training-induced increases in gray and white matter in the right IFG as well as increased white matter in the IFG-caudate tract, which correlated with improvements in proficiency compared to a control group. However, as with Stein et al. no correlation between pre-training gray/white matter and later proficiency gains was observed. These studies provide strong evidence that language training can induce neuroplastic changes, though they did not provide evidence that existing neural structures predicted subsequent language learning abilities. In contrast, a number of studies have identified neural predictors of enhanced learning, including greater volume in the HG [138] and greater frontal–parietal connectivity [132], both of which have been shown to increase with multilingual experience [70, 136]. It should be noted that in addition to different findings across studies, there were differences in methodology (e.g., training periods ranging from 1 day to 5 months) and conceptual scope (e.g., assessments of syntactic versus linguistic pitch learning). This variability highlights the need to systematically consider the interactions among linguistic, contextual, and neurocognitive factors in order to understand how language acquisition shapes the brain and how particular structures support further learning.

Table 3 provides an overview of studies on the effects of bilingualism on language learning and associated neural regions.

**Table 3:** Consequences of bilingualism for language learning

	Type	Region	Effect	Task	Study
ACC	Functional	ACC/SMA	Mono > bi (activation)	L2 word learning	Bradley et al. [165]
	Structural	ACC	+ Correlation: gray matter/L2 vocabulary size (non-training)	English vocabulary test	Hosoda et al. [77]
Frontal cortex/gyrus	Functional	R DLPFC	Mono > bi (activation)	L2 word learning	Bradley et al. [165]
	Structural	IFG	+ Correlation: gray & white matter/L2 vocabulary size (non-training & training) training > control (gray & white matter)	English vocabulary test	Hosoda et al. [77]
		Frontal lobe	Bi > mono (white matter)		Olsen et al. [180]
		L IFG	Bi only: + correlation: gray matter/improvement of L2 proficiency	L2 proficiency	Stein et al. [185]
		IFG; L MFG	Interpreters > control (CT change from T1 to T2)	L2 proficiency	Mårtensson et al. [139]
Temporal cortex/gyrus	Structural	STG/R MTG	+ Correlation: gray matter/L2 vocabulary size (non-training)	English vocabulary test	Hosoda et al. [77]
		L temporal lobule	Bi > mono (gray matter); mono ≠ bi (− correlation: bilingualism/effects of aging)	Picture naming	Abutalebi et al. [181]
		Temporal pole	Mono ≠ bi (− correlation: cortical thickness/aging; mono only)		Olsen et al. [180]
		Temporal lobe	Bi > mono (white matter)		Olsen et al. [180]
		STG	Interpreters > control (CT change from T1 to T2)	L2 proficiency	Mårtensson et al. [139]
Parietal cortex/gyrus	Functional	L IPL	Bi only: + correlation gray matter/linguistic competence & cognitive control	ANT, language competence test	Della Rosa et al. [178]
	Structural	IPL	Bi > mono (gray matter); mono ≠ bi (− correlation: RIPL gray matter/age, mono only); higher > lower proficiency (LIPL gray matter); greater > less exposure (RIPL gray matter)	Vocabulary/linguistic background measures	Abutalebi et al. [78, 179]
		pSMG	Multi > bi (gray matter density)	Lexical decision	Grogan et al. [177]
		pSMG	Bi > mono (gray matter); bi only: (+ correlation: gray matter/L2 proficiency)	L2 proficiency	Mechelli et al. [170]
Subcortical	Functional	Putamen	Bi ≠ mono (bi right putamen, mono both)	Proficiency tests	Cherodath et al. [166]
		Putamen	Bi > mono (activation)	L2 word learning	Bradley et al. [165]
		L CN	Mono > bi (activation)	L2 word learning	Bradley et al. [165]
	Structural	CN	+ Correlation: gray matter/L2 vocabulary size (non-training)	English vocabulary test	Hosoda et al. [77]
		Putamen, thalamas, globus pallidus	Bi > mono (expansion), correlation between immersion L2 and structure, not proficiency, in sequential bilinguals	Proficiency test	Pliatsikas et al. [86]
Multiple/other	Functional		Bi ≠ mono (bis showed native-like EEG responses at low proficiency of artificial language when monos did not, bis better RT and accuracy, reached proficiency sooner than monos)	Learning Brocanto2 language	Grey et al. [164]
	Structural	Frontal/temporal/parietal and occipital/temporal/parietal	Bi > mono (write matter connectivity in sub-networks)		García-Pentón et al. [182]
		L IFOF, AC-OL	Simultaneous bi > mono & sequential bi (white matter; IFOF) Simultaneous bi < mono (white mater, AC-OL)		Mohades et al. [92]
		R IFG/caudate	+ Correlation: white matter connectivity/L2 vocabulary size (non-training and training) training > control	English vocabulary test	Hosoda et al. [77]
		L IFOF	Simultaneous bi > monolinguals (white matter)		Mohades et al. [93]
		R IFOF, anterior thalamic radiation	Mono > bi (white matter)	Reading test	Cummine and Boliek [98]
		Hippocampus	Interpreters > control (volume change from T1 to T2)	L2 proficiency	Mårtensson et al. [139]

Summary of functional and structural effects of bilingual experience for tasks and neural regions associated with language learning.

In sum, bilingual experience can lead to a number of structural changes to language-related brain areas, including the LIPC, LIFG, and LATL, as well as white matter tracts connecting language-relevant regions. While it is yet unclear whether such changes can account for bilinguals’ improved language learning, people with multilingual experience often develop neural characteristics associated with better language ability in general. Similarly, bilingual experience can enhance cognitive functions that support language acquisition, such as phonological working memory and the ability to inhibit interference from known languages. It is therefore possible that the greater capacity to learn new languages brings the effects of bilingualism full circle: the need to manage greater linguistic competition is likely at the origin of numerous neurocognitive changes that may ultimately make it easier for bilinguals to acquire and control more competitors.

## Conclusion

A lifetime of managing multiple linguistic systems can have dramatic effects on both the function and structure of the bilingual neural architecture. Perhaps most surprising is the discovery that such changes can develop with relatively brief amounts of exposure to another language, highlighting the incredible plasticity of the human brain even into adulthood. The increased demands of controlling competition from candidates of multiple languages have been shown to alter how bilinguals engage in high-level processes such as executive control as well as low-level perceptual encoding of sound, including in the brainstem. Furthermore, we provide evidence that these effects are likely related, such that top-down attentional control may in fact contribute to how lower-level sensory functions operate. These often-beneficial changes at various levels of processing may, in turn, confer advantages for language learning.

The human brain is comprised of highly interactive networks that adapt to serve multiple functions. It would therefore be useful to consider the neural consequences of language experience in a similarly interrelated and comprehensive manner by studying the reciprocal relationships between different language inputs and levels of processing. Given that multilingualism is increasingly the norm rather than the exception, any model of our linguistic capacity would be incomplete without accounting for how the brain accommodates multiple languages and the subsequent changes that ripple throughout the neurocognitive system. While a number of studies have investigated the effects of bilingualism on one, or at most, two functions at a time (for example, cognitive control and speech perception, or cognitive control and language learning), even greater integration of tasks tapping into different processes could offer a more unified view. By examining the impact of multilingualism at multiple levels of processing, future work may further illuminate the interconnected and cascading effects of language experience that result in widespread consequences for cognition and the brain.

## Abbreviations

ABR: auditory brainstem response
ACC: anterior cingulate cortex
AC-OL: anterior third of corpus callosum
AF: arcuate fasciculi
Amg: amygdala
ATL: anterior temporal lobe
BAPSS: bilingual anterior to posterior and subcortical shift
BEPA: bilingual executive processing advantage
BICA: bilingual inhibitory control advantage
Cb: cerebellum
CC: corpus callosum
CN: caudate nucleus
CRN: correct response negativity
DLPFC: dorsolateral prefrontal cortex
EEG: electroencephalogram
ERN: error-related negativity
ERP: event-related potential
FFR: frequency following response
fMRI: functional magnetic resonance imaging
HG: Heschel’s gyrus
Hip: hippocampus
IFG: inferior frontal gyrus
IFOF: inferior frontal-occipital fasciculi
ILF: inferior longitudinal fasciculi
IOG: inferior occipital gyrus
IPC: inferior parietal cortex
IPL: inferior parietal lobe
ITG: inferior temporal gyrus
L: left
L2: second language
L3: third language
LOC: lateral occipital cortex
MEG: magnetoencephalography
MFG: middle frontal gyrus
MOG: middle occipital gyrus
MTG: middle temporal gyrus
PFC: prefrontal cortex
pSMG: posterior supramarginal gyrus
R: right
SMA: supplementary motor area
SFG: superior frontal gyrus
SLF: superior longitudinal fasciculi
SOG: superior occipital gyrus
STG: superior temporal gyrus
STS: superior temporal sulcus
UF: uncinate fasciculi
WM: white matter
